# A compass in the challenge: the CALLY index as a prognostic biomarker in advanced cholangiocarcinoma treated with chemoimmunotherapy: a retrospective propensity score-matched cohort study

**DOI:** 10.3389/fimmu.2026.1713495

**Published:** 2026-02-24

**Authors:** Runjia Fan, Zijian Lv, Tiance Wang, Wei Chen, Weidong Han, Qian Mei

**Affiliations:** 1 Department of Bio-Therapeutic, The First Medical Center, Chinese People’s Liberation Army General Hospital, Beijing, China; 2 Department of Hematology, General Hospital of Northern Theater Command, Shenyang, Liaoning, China; 3 Department of Pathology, The First Medical Center, Chinese People’s Liberation Army General Hospital, Beijing, China

**Keywords:** advanced cholangiocarcinoma, CALLY, chemoimmunotherapy, prognostic value, propensity score matching

## Abstract

**Introduction:**

The management of advanced cholangiocarcinoma (CCA) remains a clinical challenge. Prognostic biomarkers are needed to guide treatment decisions. The C-reactive protein–albumin–lymphocyte (CALLY) index reflects nutritional, immune, and inflammatory status and has shown prognostic value in other cancers. However, its role in CCA patients receiving chemoimmunotherapy is unexplored.

**Methods:**

We conducted a retrospective propensity score-matched (PSM) cohort study involving advanced CCA patients who were treated with chemoimmunotherapy. Participants were stratified into high- or low-CALLY groups based on an optimal cut-off value of 1.42. PSM (1:1) was applied to balance baseline covariates. Overall survival (OS) and progression-free survival (PFS) were compared between groups using Kaplan-Meier analysis, and Cox regression analysis was employed to identify prognostic factors. The prognostic models underwent comprehensive internal validation, including bootstrap resampling (1,000 iterations) for calibration and discrimination assessment. Health-related quality of life (HRQoL) was assessed using a mixed model for repeated measures.

**Results:**

After 1:1 propensity matching, 55 patients were retained in each group, with balanced baseline characteristics. The high-CALLY group exhibited significantly longer median OS (13.00 months vs. 11.50 months; P = 0.019) and PFS (7.50 months vs. 6.00 months; P = 0.020). Cox analysis confirmed the CALLY index as a valuable prognostic factor for both OS (hazard ratio (HR), 0.68; 95% confidence interval (CI), 0.50 to 0.93; P = 0.014) and PFS (HR, 0.70; 95% CI, 0.58 to 0.85; P < 0.001). Internal validation demonstrated good model performance, with optimism-corrected C-indices of 0.704 for OS and 0.716 for PFS. Furthermore, patients with a high CALLY index showed significantly slower deterioration in HRQoL from week 18 onward (P < 0.05).

**Conclusion:**

The CALLY index is a robust prognostic biomarker for advanced CCA patients undergoing chemoimmunotherapy, associated with significantly improved survival and better-preserved quality of life. Its integration into clinical practice could enhance risk stratification and facilitate personalized treatment strategies.

## 1 Introduction

Cholangiocarcinoma (CCA) is a highly lethal malignant tumor originating from the biliary system. Based on the anatomical site of origin, CCA can be classified into three subtypes: intrahepatic cholangiocarcinoma (iCCA), perihilar cholangiocarcinoma (pCCA), and distal cholangiocarcinoma (dCCA). The latter two subtypes are collectively termed extrahepatic cholangiocarcinoma (eCCA) (1). CCA progresses aggressively, frequently leading to cachexia and inanition, consequently resulting in rapid deterioration in performance status. Globally, both the incidence and mortality of CCA have continued to rise (1–3). Up to 85% of patients are diagnosed at an advanced stage, thereby precluding curative treatment options (4). Consequently, the 5-year survival rate remains poor, ranging from approximately 3% to 13% (4–8).

Systemic chemotherapy remains the standard treatment for advanced stage CCA. In the past few years, the combination of gemcitabine and cisplatin has been the front-line standard regimen for the treatment of advanced CCA, yielding a median survival of approximately 1 year (9, 10). More recently, the TOPAZ-1 (11, 12) and KEYNOTE-966 (13) trials have demonstrated significantly improved survival with the addition of a programmed death-ligand 1 (PD-L1) or programmed cell death protein 1 (PD-1) inhibitor to chemotherapy. This regimen has consequently emerged as a new front-line standard (8). By contrast, targeted therapies have thus far shown limited efficacy in the majority of CCA patients (14). It is worth noting that even in targeted therapy, such as FGFR inhibitors, real-world evidence suggests moderate efficacy with a median progression-free survival of 5.8 months in heavily pretreated patients, though selection bias and patient heterogeneity may limit generalizability (15). Patient outcomes remain heterogeneous and depend on multiple factors including tumor biology, genetics, nutritional status, and immune-inflammatory responses. The majority of prognostic tools used to predict outcomes among patients with CCA rely on staging systems, as well as markers such as carcinoembryonic antigen (CEA) and carbohydrate antigen (CA) 19-9, which correlate with tumor aggressiveness; however, these tools demonstrate limited accuracy for personalized prognostication and treatment allocation. Consequently, novel prognostic biomarkers are critically needed to inform therapeutic approaches and surveillance strategies aimed at improving patient outcomes.

Immune-, Inflammation-, and nutrition-based indices—including the neutrophil-to-lymphocyte ratio (NLR) (16), modified Glasgow Prognostic Score (mGPS) (17), Prognostic Nutritional Index (PNI) (18), and cachexia index (CXI) (19)—have gained recognition for their cost-effectiveness and prognostic utility in CCA. Building upon these, Iida et al. (20) developed the CRP-Albumin-Lymphocyte (CALLY) index, a non-invasive biomarker integrating albumin (nutrition), absolute lymphocyte count (immune status), and C-reactive protein (CRP; inflammation) levels. The CALLY index is calculated as: albumin (g/dL) × absolute lymphocyte count (cells/µL)/[CRP (mg/dL) × 10^4^]. However, its prognostic value has not yet been validated in advanced CCA patients receiving chemoimmunotherapy. Moreover, as a composite biomarker, the CALLY index reflects a multidimensional physiological state, while this population often presents with heterogeneous clinical characteristics. This complexity may introduce confounding effects, complicating the interpretation of the association between the CALLY index and clinical outcomes. Therefore, to robustly evaluate the prognostic role of the CALLY index while controlling for confounding bias, we conducted a retrospective propensity score-matched (PSM) cohort study in this setting.

## 2 Methods

### 2.1 Study design and oversight

We conducted a retrospective PSM cohort study at the Chinese People’s Liberation Army (PLA) General Hospital involving patients with advanced CCA receiving chemoimmunotherapy. Participants were stratified into low- and high-CALLY groups based on an optimal cut-off value of the CALLY index. This study is reported in accordance with the Strengthening the Reporting of Observational Studies in Epidemiology (STROBE) guidelines (21). The study involving humans was approved by the Ethics Committee of the Chinese PLA General Hospital (Ethical Approval No.: 2023-X19-93). The study was conducted in accordance with the local legislation and institutional requirements. The participants provided their written informed consent to participate in this study. All medical records were anonymized and de-identified prior to analysis. All authors attest to the completeness and veracity of the data.

### 2.2 Participants

Patients with advanced CCA (including iCCA, pCCA, and dCCA) who received chemoimmunotherapy (without concomitant targeted therapy) at the Chinese PLA General Hospital between August 2017 and December 2024 were retrospectively enrolled. The front-line regimen consisted of gemcitabine plus cisplatin combined with a PD-1 or PD-L1 inhibitor. For later-line therapy, although the chemotherapy backbone could vary, the principle of combining it with an immune checkpoint inhibitor was consistently maintained. The inclusion criteria were: age ≥18 years; histologically confirmed CCA diagnosis; unresectable or metastatic disease at initial diagnosis, or recurrent disease after curative-intent surgery in patients who were deemed unsuitable for further surgical intervention and therefore proceeded directly to systemic therapy; comprehensive hematologic and biochemical profiling within 7 days prior to initiating chemoimmunotherapy; and availability of ≥ 1 post-baseline disease assessment. Exclusion criteria included active or documented autoimmune or inflammatory disorders that could materially influence CRP-based indices (including biliary obstruction or infection, drainage, hepatitis B or C virus infection); previous liver transplantation; significant comorbidities (concurrent malignancies, NYHA class III or IV cardiac dysfunction, Child-Pugh B or C hepatic insufficiency, or renal impairment with an estimated glomerular filtration rate (eGFR) <30 mL/min/1.73m²); or other serious medical conditions.

### 2.3 Variables and outcomes

The following baseline data were collected from all participants: sex, age, body mass index (BMI), smoking status, treatment line, tumor location, ECOG performance status, and laboratory parameters. The latter included CA 19–9 and the CALLY index, both of which were measured within one week prior to the initiation of chemoimmunotherapy. The CALLY index was calculated as: Albumin (g/dL) × Absolute Lymphocyte Count (cells/µL)/[C-reactive Protein (mg/dL) × 10^4^]. All laboratory values were obtained using standard assays, and unit conversions were verified prior to index calculation. Treatment exposure variables were summarized descriptively in the matched cohort, including ICI class and agent, concurrent chemotherapy backbone, induction cycles, total ICI cycles, maintenance strategy, and subsequent therapies (**Supplementary Table S2**). Tumor measurements were performed by computed tomography (CT) or magnetic resonance imaging (MRI) scans at baseline and every two treatment cycles until progressive disease (PD). PD was defined as the appearance of new measurable lesions, unequivocal progression of non-target lesions, an absolute increase of ≥ 5 mm, or a ≥ 20% increase in the sum of diameters of target lesions. Objective disease assessments were conducted by the investigators using all available post-baseline scans in accordance with RECIST version 1.1.

The primary outcome was overall survival (OS), defined as the time from the initiation of chemoimmunotherapy to death from any cause. Patients without an event were censored on the last date they were confirmed to be alive. Secondary outcomes included progression-free survival (PFS) and health-related quality of life (HRQoL). PFS was defined as the time from treatment initiation to radiologically confirmed PD, mortality, or the last tumor evaluation. HRQoL was assessed every two treatment cycles using a visual analogue scale (VAS). On the VAS, participants rated their current health state by marking a line between 0 (“the worst health you can imagine”) and 100 (“the best health you can imagine”).

### 2.4 Statistical methods

Statistical analyses were performed using R software (version 4.3.0) and SAS software (version 9.4). Categorical variables were presented as numbers and percentages. The normality of continuous variables was assessed using the Shapiro-Wilk test. Based on the test results, normally distributed variables were presented as mean ± standard deviation, while non-normally distributed variables were summarized as median with interquartile range (interquartile range, 25th–75th percentile). The optimal cut-off value of the CALLY index for OS was determined using an automated grid search algorithm that maximized the log-rank test statistic, implemented via the “surv_cutpoint” function from the “survminer” R package. Its stability was evaluated via nonparametric bootstrap resampling (1,000 iterations), summarizing the empirical distribution (median and 2.5th–97.5th percentile).

To minimize selection bias and potential confounding in outcome comparisons, PSM was performed using a 1:1 greedy nearest-neighbor matching method without replacement. Propensity scores were estimated via multivariable logistic regression including pre-specified covariates, excluding outcome variables. The model included the following covariates: age, sex, BMI, CA 19-9, treatment line (front-line vs. later-line), tumor location (iCCA vs. eCCA), and ECOG performance status (≤1 vs. >1). Matched pairs were created by matching high- and low-CALLY participants using calipers with a width of 0.15 standard deviations of the logit propensity score. Covariate balance before and after PSM was assessed using standardized mean differences (SMDs), with |SMD| < 0.10 indicating good balance. Balance diagnostics were visualized using a Love plot. Propensity score distributions were inspected to evaluate overlap (common support) between groups before and after matching.

OS and PFS were estimated using the Kaplan-Meier method, and differences between groups were compared with the log-rank test. Univariate and multivariate Cox proportional hazards models were employed to identify risk factors associated with OS. Results were reported as hazard ratios (HRs) with 95% confidence intervals (CIs). Variables with a univariate P value < 0.10 or considered clinically relevant were included in the multivariate Cox regression analysis. To mitigate potential overfitting from data-driven dichotomization, CALLY was modeled as a continuous predictor in Cox proportional hazards models (reported per 1-unit increase). In the matched cohort, Cox proportional hazards models were fitted accounting for the paired nature of 1:1 matching by using robust (sandwich) standard errors clustered on the matched pair identifier (MatchID). This approach provides valid inference under within-pair correlation after matching. Potential nonlinearity was assessed using restricted cubic splines (4 knots), and quantile-based sensitivity analyses (quartiles). The proportional hazards assumptions were verified using Schoenfeld residual–based tests to ensure model validity. Subsequently, we performed comprehensive internal validation using bootstrap resampling (1,000 iterations) to estimate model optimism and obtain optimism-corrected concordance indices (C-indices). Model calibration was assessed by constructing calibration curves for 1-year OS and 6-month PFS using bootstrap bias-correction. Finally, we evaluated the model’s discriminative ability over time by conducting time-dependent receiver operating characteristic (ROC) analyses at clinically relevant time points (12 and 18 months for OS; 6 and 9 months for PFS). To address potential calendar-time-related confounding over the whole enrollment period, we defined treatment era by index regimen start year (<2021 vs ≥2021) and performed Cox models stratified by treatment era for OS and PFS in the matched cohort (**Supplementary Table S3**).

HRQoL was assessed using VAS, with change from baseline to week 30 defined as the endpoint. This time point was selected as the latest satisfying pre-specified thresholds to minimize attrition bias: ≥ 80% for completion rate (the proportion of participants who completed the assessment at the specific time point) and ≥ 90% for compliance rate (the proportion of participants who completed the questionnaire among those expected to do so at that time point). Between-group differences in least squares mean (LSM) changes from baseline in VAS were compared using a mixed model for longitudinal repeated measures, with fixed effects including group, time, and the group × time interaction. The within-subject correlation over time was modeled using an unstructured covariance matrix (TYPE=UN). Model parameters were estimated using restricted maximum likelihood (REML), and inference from this likelihood-based MMRM was made under the missing-at-random (MAR) assumption. For all endpoints, between-group differences in LSM changes were estimated along with 95% confidence intervals (CIs) using SAS PROC MIXED. Other comparisons between groups were performed using independent-sample t-tests (for normally distributed variables) or the Mann-Whitney U test (for non-normally distributed variables) for continuous variables, and chi-square or Fisher’s exact tests for categorical variables, as appropriate. A two-sided P value < 0.05 was considered statistically significant for all analyses.

## 3 Results

### 3.1 Patients and baseline characteristics

Between August 2017 and December 2024, a total of 251 patients who received chemoimmunotherapy were screened for eligibility, of whom 29 were excluded. The optimal cut-off value of the CALLY index for OS was 1.42, which was determined using an automated grid search algorithm that maximized the log-rank test statistic. Bootstrap resampling demonstrated that the cutpoint estimate was reasonably stable (original 1.42; 2.5th–97.5th percentile: 1.38–1.45) (**Supplementary Figure S1**). Using an optimal cut-off value of 1.42 for the CALLY index based on OS, the remaining 222 patients (mean age, 62.68 ± 9.81 years; 121 males and 101 females; 208 with metastatic disease and 14 with locally advanced disease) were stratified into low- or high-CALLY groups. A PSM was then applied to balance baseline characteristics between the two groups, yielding a final matched cohort of 110 patients (55 per group) for subsequent analyses.

**Figure 1:**
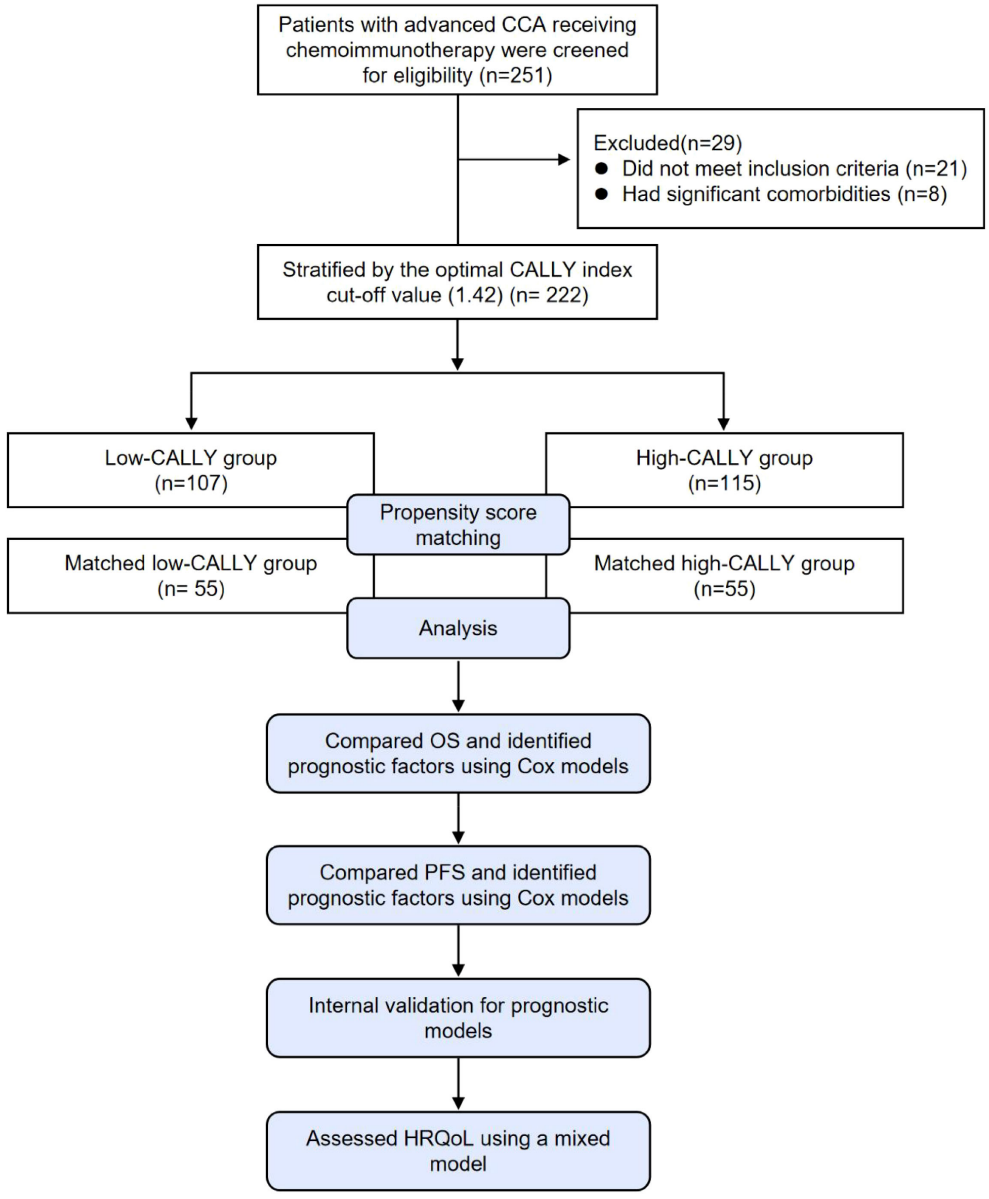
Flowchart of patient selection, propensity score matching, and subsequent analysis. A total of 251 patients with advanced cholangiocarcinoma receiving chemoimmunotherapy were initially screened. After excluding 29 patients, 222 patients were stratified into high- and low-CALLY groups based on the optimal cut-off value of 1.42. Propensity score matching was then performed to balance baseline characteristics, resulting in 55 matched pairs (n=55 per group). The final matched cohorts were compared for OS and PFS using Kaplan-Meier and Cox regression analyses. Internal validation for the prognostic models was subsequently conducted. HRQoL was assessed using a mixed model. CALLY, CRP-albumin-lymphocyte index; OS, overall survival; PFS, progression-free survival; HRQoL, health-related quality of life.

Before matching, patients in the high-CALLY group were significantly younger than those in the low-CALLY group. Other variables that might influence prognosis—including CA 19–9 level, treatment line, tumor location, and ECOG performance status—also exhibited numerical differences, although these did not reach statistical significance. After PSM, all baseline characteristics were well balanced between the two groups as assessed by SMDs (**Table 1**) and visualized by the Love plot (**Supplementary Figure S3**). Overall, covariate imbalance was substantially reduced after matching, and propensity score distributions demonstrated improved overlap (common support) in the matched cohort (**Supplementary Figures S4A, B**). A small residual imbalance remained for age (SMD ≈ 0.12), which was further accounted for in the post-matching Cox models. **Figure 1** outlines the patient selection process and illustrates how PSM was applied to form two comparable cohorts of 55 patients in each group, thereby ensuring balanced baseline characteristics for robust comparative analysis.

**Table 1:** Clinical characteristics of patients before and after PSM.

Characteristics	Before PSM					After PSM				
	Total (n=222)	Low-CALLY group (n=107)	High-CALLY group (n=115)	SMD	P value	Total (n=110)	Low-CALLY group (n=55)	High-CALLY group (n=55)	SMD	P value
Age, years (Mean ± SD)	62.68 ± 9.81	64.17 ± 9.46	61.30 ± 9.96	-0.30	0.029	62.61 ± 9.17	63.18 ± 8.85	62.04 ± 9.52	-0.12	0.515
Sex, n (%)				0.13	0.321				0	1.000
Male	121 (54.50)	62 (57.94)	59 (51.30)			60 (54.55)	30 (54.55)	30 (54.55)		
Female	101 (45.50)	45 (42.06)	56 (48.70)			50 (45.45)	25 (45.45)	25 (45.45)		
Smoke, n (%)				-0.17	0.214				0	1.000
Yes	84 (37.84)	36 (33.64)	48 (41.74)			38 (34.55)	19 (34.55)	19 (34.55)		
No	138 (62.16)	71 (66.36)	67 (58.26)			72 (65.45)	36 (65.45)	36 (65.45)		
BMI (Mean ± SD)	22.95 ± 3.00	22.86 ± 2.76	23.03 ± 3.21	0.05	0.686	22.83 ± 3.11	22.91 ± 2.89	22.75 ± 3.34	-0.06	0.782
Tumor location, n (%)				0.13	0.334				0	1.000
iCCA	84 (37.84)	37 (34.58)	47 (40.87)			34 (30.91)	17 (30.91)	17 (30.91)		
eCCA	138 (62.16)	70 (65.42)	68 (59.13)			76 (69.09)	38 (69.09)	38 (69.09)		
ECOG, n (%)					0.269					1.000
≤1	141 (63.51)	64 (59.81)	77 (66.96)			40 (36.36)	20 (36.36)	20 (36.36)		
>1	81 (36.49)	43 (40.19)	38 (33.04)			70 (63.64)	35 (63.64)	35 (63.64)		
CA 19-9 (Mean ± SD)	330.62 ± 595.88	391.42 ± 648.21	274.06 ± 539.39	-0.20	0.143	221.24 ± 313.23	221.77 ± 294.03	220.72 ± 334.04	-0.002	0.986
Treatment line, n (%)				0.15	0.280				0	1.000
Front-line	110 (49.55)	49 (45.79)	61 (53.04)			52 (47.27)	26 (47.27)	26 (47.27)		
Later-line	112 (50.45)	58 (54.21)	54 (46.96)			58 (52.73)	29 (52.73)	29 (52.73)		

PSM, propensity score matching; CALLY, CRP-albumin-lymphocyte index; SMD, standardized mean difference; SD, standard deviation; BMI, body mass index; iCCA, intrahepatic cholangiocarcinoma; eCCA, extrahepatic cholangiocarcinoma; CA 19-9, carbohydrate antigen 19-9.

### 3.2 Primary outcomes

**Figure 2:**
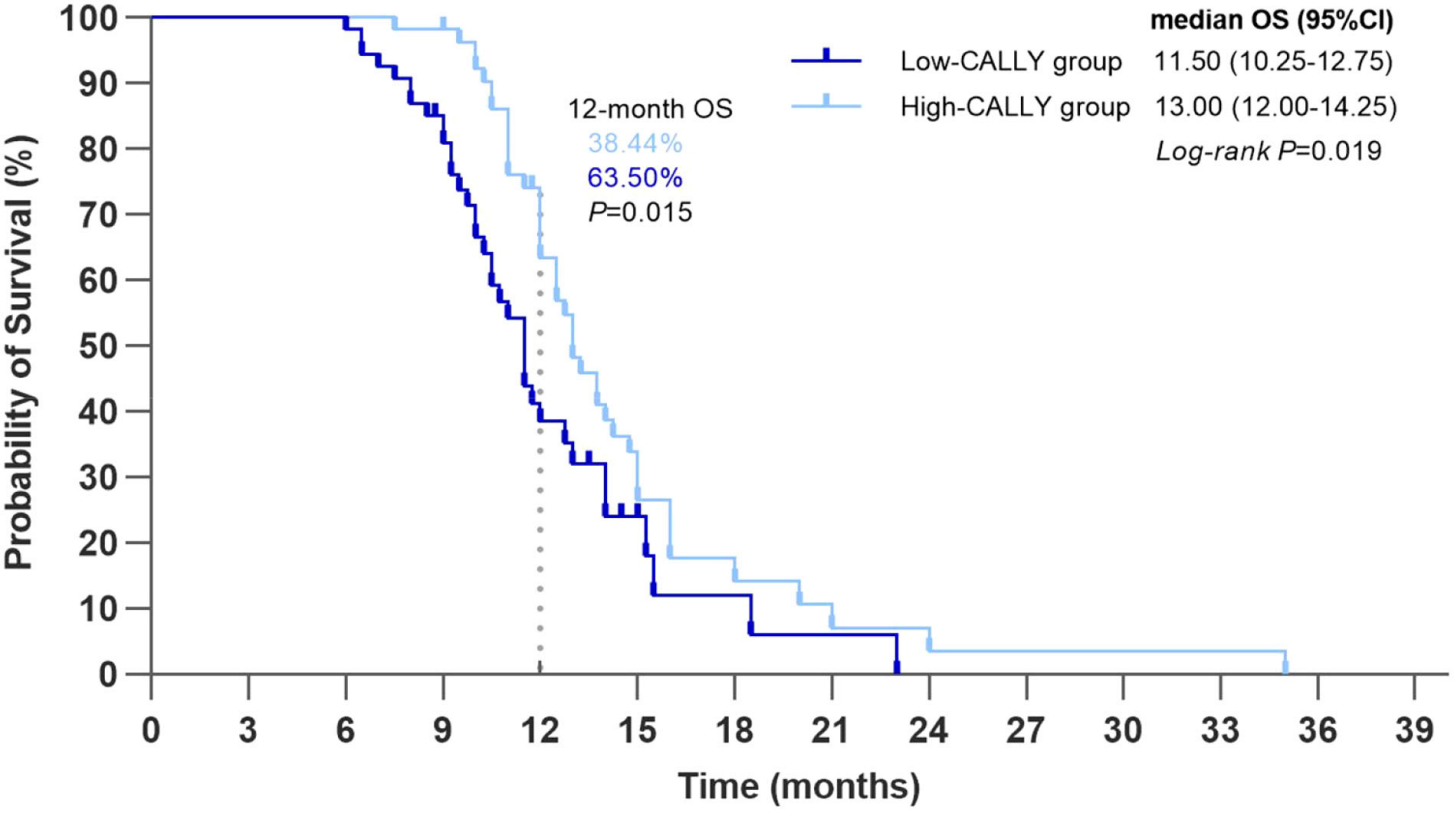
Kaplan-Meier curves for OS. Kaplan–Meier estimates for high CALLY and low CALLY groups. Median OS: 13.00 months (95% CI, 12.00–14.25) vs 11.50 months (95% CI, 10.25–12.75); log-rank P = 0.019. Estimated 12-month OS rate: 63.50% vs 38.44%; P = 0.015. OS, overall survival; CALLY, CRP-albumin-lymphocyte index; CI, confidence interval.

By December 2024, the Kaplan-Meier analysis of the PSM cohort showed significantly longer median OS in the high-CALLY group compared with the low-CALLY group (13.00 months [95% CI, 12.00 to 14.25] vs. 11.50 months [95% CI, 10.25 to 12.75]; log-rank P = 0.019). The estimated 12-month OS rate was also significantly higher in the high-CALLY group (63.50% [95% CI, 50.00 to 77.00] vs. 38.44% [95% CI, 23.49 to 53.39]; P = 0.015) (**Figure 2**). Restricted cubic spline analyses for CALLY suggested a statistically significant overall association with OS and evidence of nonlinearity (overall P = 0.006; P for nonlinearity = 0.018) (**Supplementary Figure S2**). Quantile-based analyses yielded consistent directionality across quartiles with an ordinal trend test (P = 0.065, **Supplementary Table S1**).

**Figure 3:**
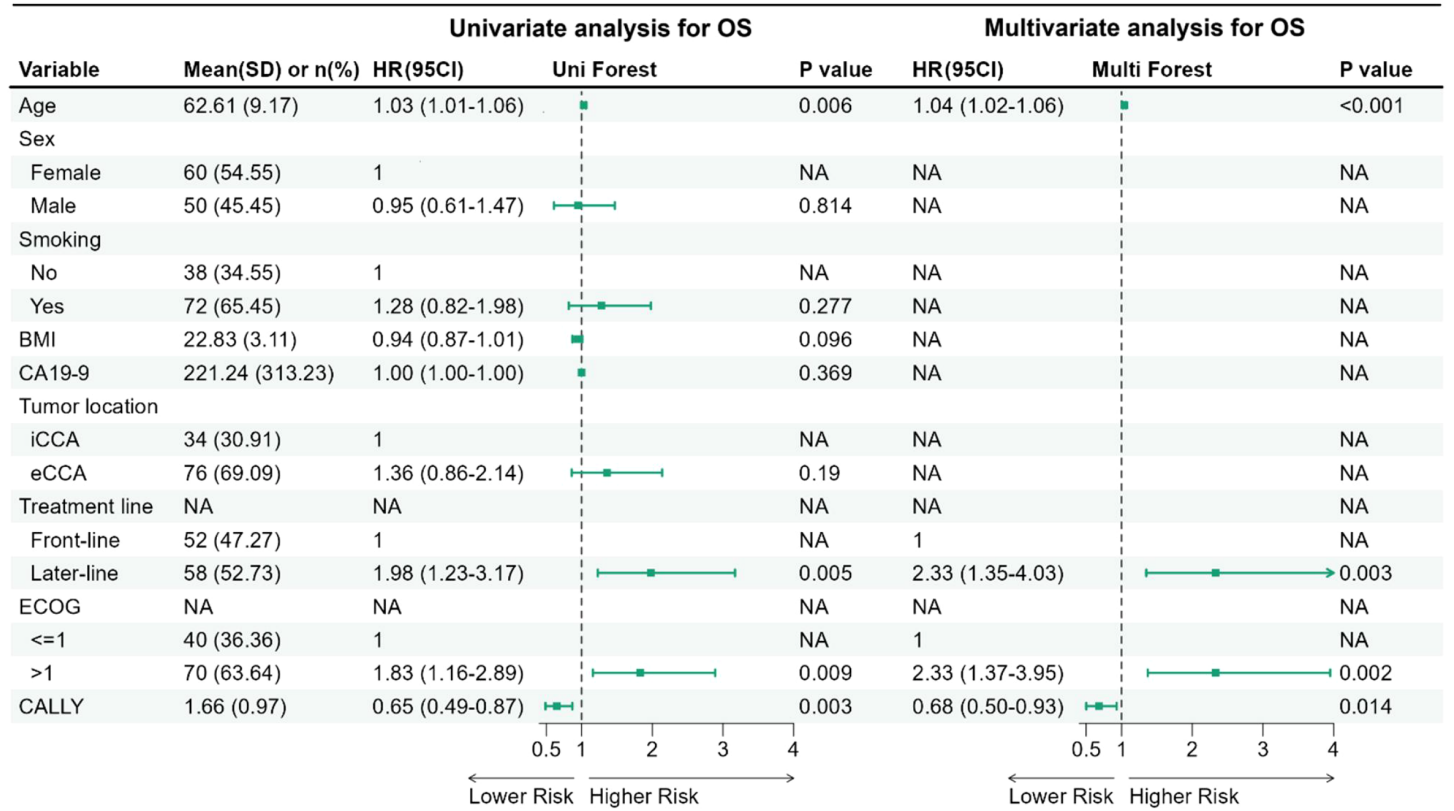
Univariate and multivariate Cox proportional hazards models for OS. Forest plot summarizing hazard ratios (HRs) and 95% confidence intervals (CIs) from univariate and multivariable Cox proportional hazards models for OS in the 1:1 propensity score–matched cohort. CALLY was modeled as a continuous predictor (per 1-unit increase). To account for the matched design, variance estimation used robust (sandwich) standard errors clustered by matched pair (MatchID) (pair-robust SE). The multivariable model was constructed using stepwise selection from prespecified baseline covariates; covariates not retained in the final multivariable model are shown as NA. OS, overall survival; SD, standard deviation; CI, confidence interval; BMI, body mass index; iCCA, intrahepatic cholangiocarcinoma; eCCA, extrahepatic cholangiocarcinoma; CA 19-9, carbohydrate antigen 19-9; CALLY, CRP-albumin-lymphocyte index; NA, not applicable.

In the univariate Cox analysis, Cox models were fitted with robust standard errors clustered by matched pair to account for within-pair correlation. Increased age (HR, 1.03; 95% CI, 1.01 to 1.06; P = 0.006), later-line treatment (HR, 1.98; 95% CI, 1.23 to 3.17; P = 0.005), and an ECOG status > 1 (HR, 1.83; 95% CI, 1.16 to 2.89; P = 0.009) were significantly associated with an increased risk of mortality. Conversely, a higher CALLY index was significantly associated with a lower risk of mortality (per 1-unit increase: HR, 0.65; 95% CI, 0.49 to 0.87; P = 0.003). No significant associations were observed for sex, smoking history, BMI, CA 19–9 level, or tumor location. The multivariate analysis with stepwise optimal model selection confirmed that increased age (HR, 1.04; 95% CI, 1.02 to 1.06; P = 0.006), later-line treatment (HR, 2.33; 95% CI, 1.35 to 4.03; P = 0.003), and an ECOG status > 1 (HR, 2.33; 95% CI, 1.37 to 3.95; P = 0.002) remained independent predictors of poorer OS. After adjustment for these variables, a high CALLY index remained significantly associated with a reduced risk of mortality (per 1-unit increase: HR, 0.68; 95% CI, 0.50 to 0.93; P = 0.014) (**Figure 3**).

### 3.3 Secondary outcomes

**Figure 4:**
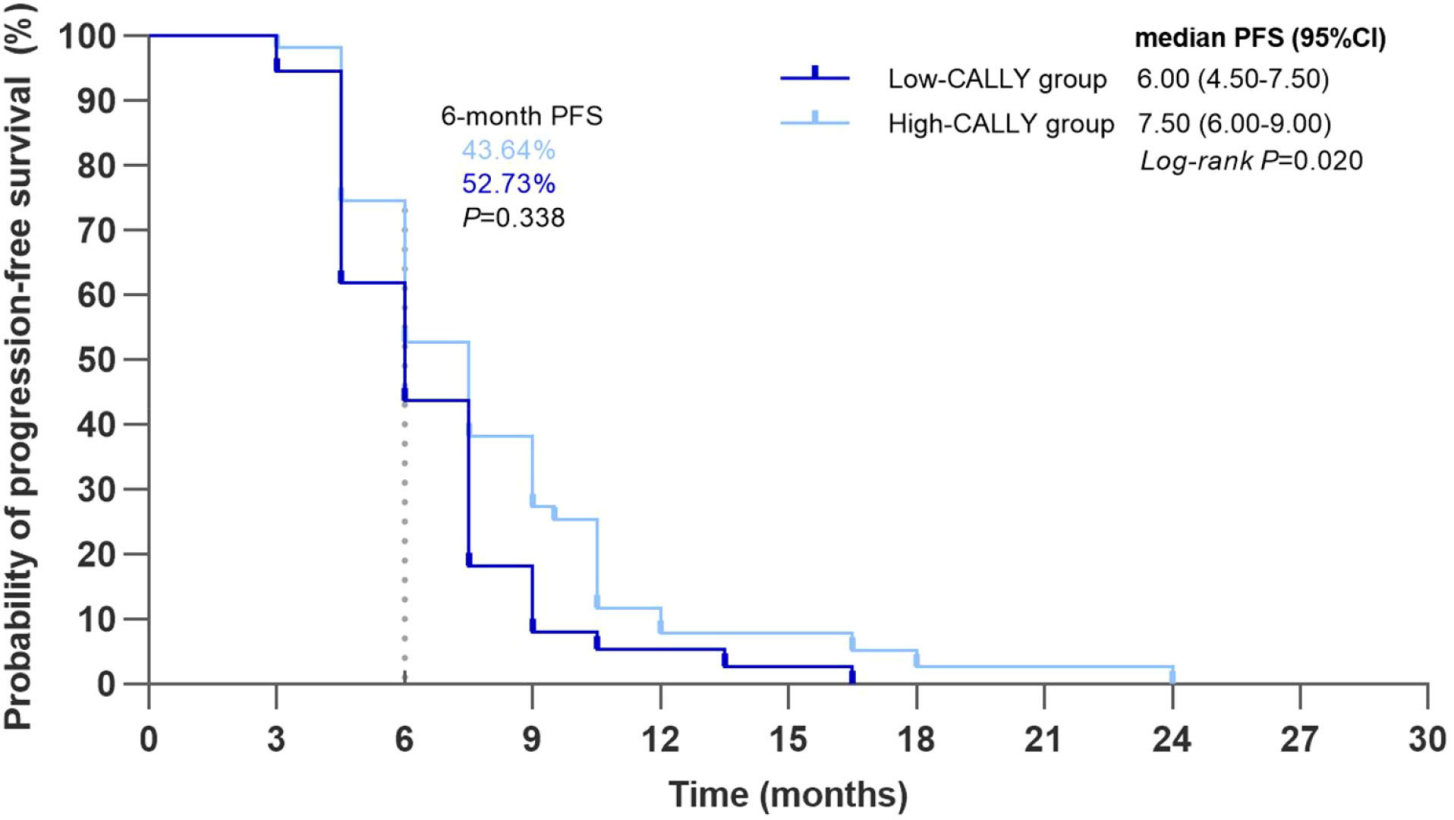
Kaplan-Meier curves for PFS. Kaplan–Meier estimates for high CALLY and low CALLY groups. Median PFS: 7.50 months (95% CI, 6.00–9.00) vs 6.00 months (95% CI, 4.50–7.50); log-rank P = 0.020. Estimated 6-month PFS rate: 52.73% vs 43.64%; P = 0.338. PFS, progression-free survival; CALLY, CRP-albumin-lymphocyte index; CI, confidence interval.

With respect to PFS, the Kaplan-Meier analysis revealed significantly longer median PFS in the high-CALLY group compared with the low-CALLY group (7.50 months [95% CI, 6.00 to 9.00] vs. 6.00 months [95% CI, 4.50 to 7.50]; log-rank P = 0.020). The 6-month PFS rate was numerically higher, though not statistically significant, in the high-CALLY group (52.73% [95% CI, 39.54 to 65.92] vs. 43.64% [95% CI, 30.53 to 56.75]; P = 0.338) (**Figure 4**).

**Figure 5:**
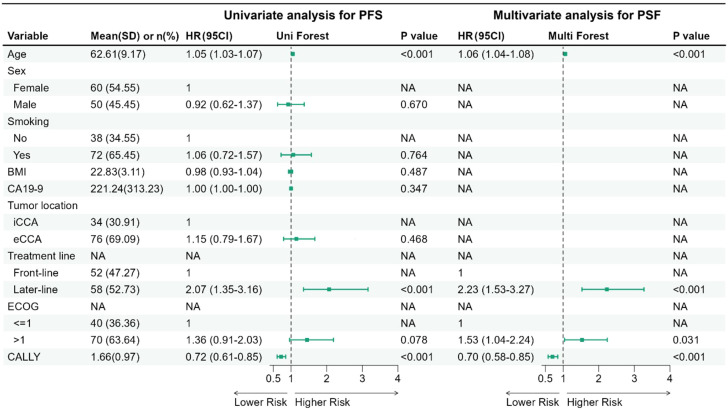
Univariate and multivariate Cox proportional hazards models for PFS. Forest plot summarizing HRs and 95% CIs from univariate and multivariable Cox proportional hazards models for PFS in the 1:1 propensity score–matched cohort. CALLY was modeled as a continuous predictor (per 1-unit increase). To account for the matched design, variance estimation used robust (sandwich) standard errors clustered by matched pair (MatchID) (pair-robust SE). The multivariable model was constructed using stepwise selection from prespecified baseline covariates; covariates not retained in the final multivariable model are shown as NA. PFS, progression-free survival; SD, standard deviation; CI, confidence interval; BMI, body mass index; iCCA, intrahepatic cholangiocarcinoma; eCCA, extrahepatic cholangiocarcinoma; CA 19-9, carbohydrate antigen 19-9; CALLY, CRP-albumin-lymphocyte index; NA, not applicable.

The univariate Cox analysis for PFS showed that increased age (HR, 1.05; 95% CI, 1.03 to 1.07; P < 0.001) and later-line treatment (HR, 2.07; 95% CI, 1.35 to 3.16; P < 0.001) were significantly associated with disease progression, whereas a higher CALLY index was associated with a reduced risk of progression (per 1-unit increase: HR, 0.72; 95% CI, 0.61 to 0.85; P < 0.001). The multivariate analysis confirmed that increased age (HR, 1.06; 95% CI, 1.04 to 1.08; P < 0.001), later-line treatment (HR, 2.23; 95% CI, 1.53 to 3.27; P < 0.001) and an ECOG status > 1 (HR, 1.53; 95% CI, 1.04 to 2.24; P = 0.031) remained independent risk factors for progression. After adjustment for these variables, a high CALLY index remained significantly associated with a reduced risk of progression (per 1-unit increase: HR, 0.70; 95% CI, 0.58 to 0.85; P < 0.001) (**Figure 5**).

### 3.4 Validation of prognostic models

**Figure 6:**
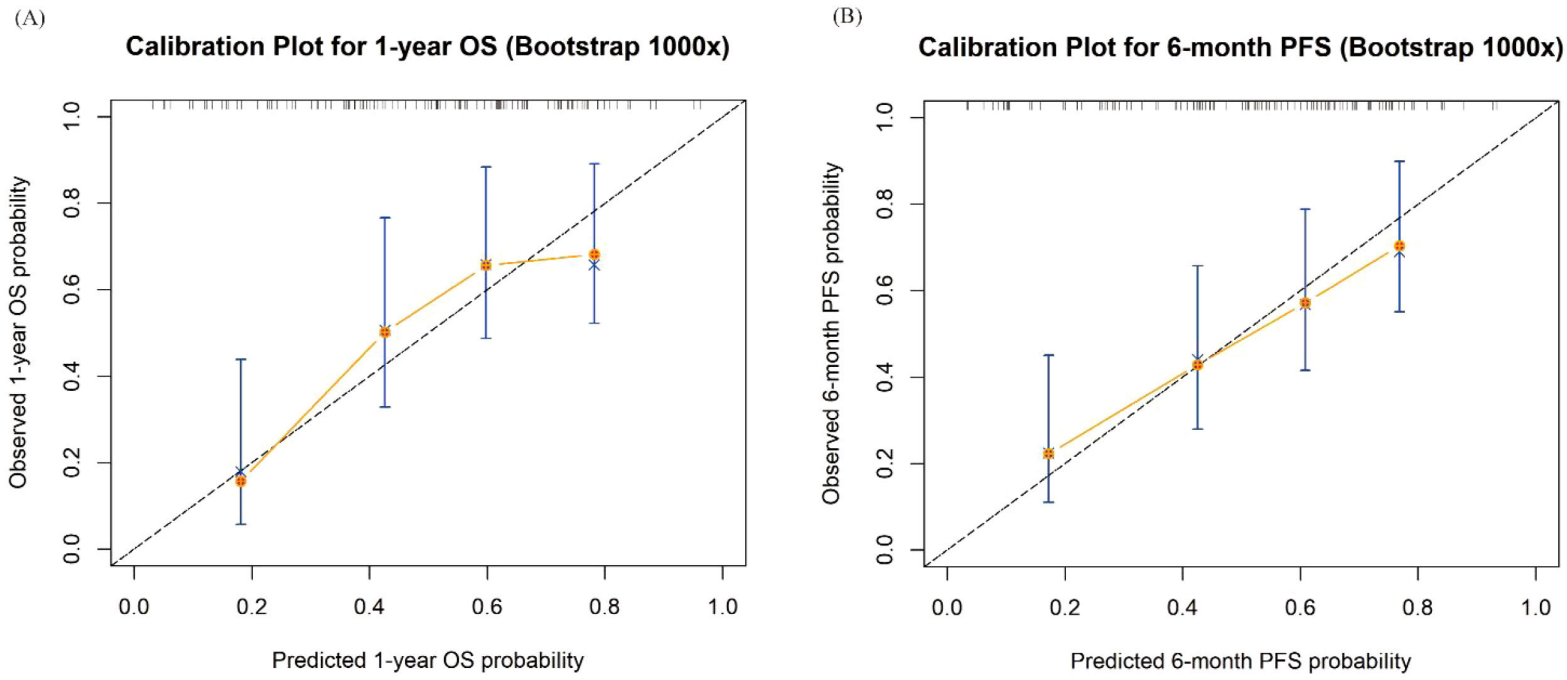
Calibration curves of the multivariate Cox model. **(A)** Calibration curve for 1-year OS; **(B)** Calibration curve for 6-month PFS. Calibration curves depict the internal validation of the model’s predictive performance using bootstrap method (1,000 iterations). The black dashed diagonal line represents the ideal reference line, where predictions would perfectly match observations. The orange solid line represents the calibration curve of the model, and the blue bars represent the 95% confidence intervals derived from bootstrap resampling. The close alignment of the calibration curves with the diagonal line in both panels **(A, B)** indicates good agreement between predicted and observed outcomes, demonstrating satisfactory calibration and predictive accuracy of the model. OS, overall survival; PFS, progression-free survival.

**Figure 7:**
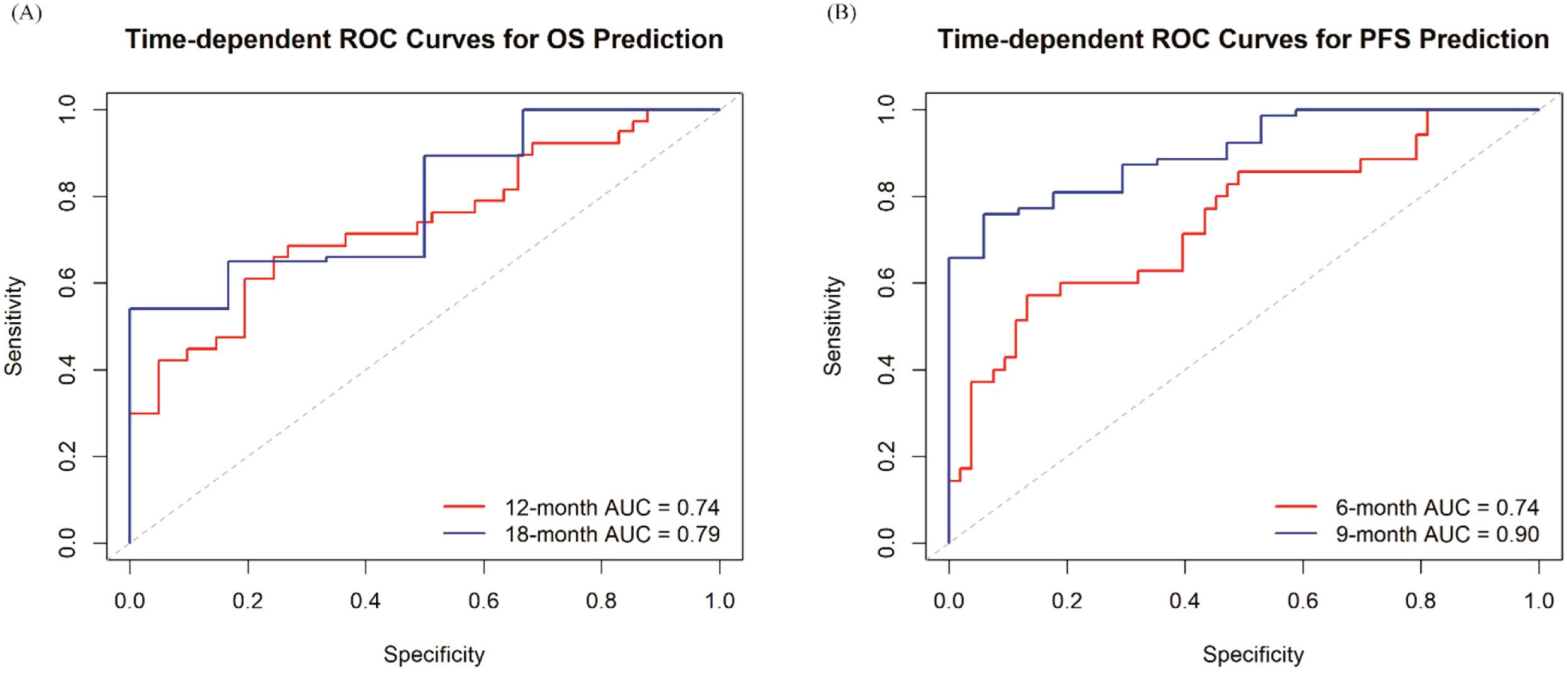
Time-dependent ROC curves of the prognostic model for predicting OS and PFS. **(A)** Time-dependent ROC curves for predicting 12-month and 18-month OS; **(B)** Time-dependent ROC curves for predicting 6-month and 9-month PFS. The AUC values are 0.74 and 0.79 for predicting 12-month and 18-month OS **(A)**, and 0.74 and 0.90 for 6-month and 9-month PFS **(B)**. The gray dashed line represents the reference line of no discrimination (AUC = 0.5). AUC values exceeding 0.7 at evaluated time points indicate good predictive accuracy of the model. OS, overall survival; PFS, progression-free survival; ROC, receiver operating characteristic; AUC, area under the curve.

Comprehensive internal validation confirmed the robustness and reliability of both prognostic models. The proportional hazards assumption was first verified using Schoenfeld residuals, which revealed no significant violations (P = 0.416 for OS; P = 0.750 for PFS). Bootstrap resampling (1,000 iterations) yielded minimal optimism estimates of 0.006 for the OS model and 0.005 for the PFS model. After optimism correction, the C-indices were 0.704 (95% CI: 0.639 to 0.791) for OS and 0.716 (95% CI: 0.657 to 0.789) for PFS, indicating good overall discrimination. The calibration curves based on the same bootstrap procedure showed close agreement between predicted and observed probabilities for 1-year OS and 6-month PFS, closely aligning with the 45-degree reference line (**Figure 6**). Furthermore, the time-dependent ROC analysis demonstrated excellent time-specific performance, with area under the curve (AUCs) of 0.74 at 12 months and 0.79 at 18 months for OS, and 0.74 at 6 months and 0.90 at 9 months for PFS, respectively (**Figure 7**), the latter indicating superior predictive accuracy at later time points. The association between CALLY and outcomes remained significant in analyses stratified by treatment era, suggesting robustness to evolving treatment practice over calendar time (**Supplementary Table S3**).

### 3.5 HRQoL outcomes

HRQoL was assessed using the VAS. Based on pre-specified thresholds for questionnaire completion (≥80%) and compliance (≥90%), week 30 was identified as the latest evaluable time point. At week 30, the VAS completion rates were 80.00% (44 of 55) in the low-CALLY group and 87.27% (48 of 55) in the high-CALLY group, with compliance rates of 91.67% (44 of 48) and 90.57% (48 of 53), respectively. Missing assessments were primarily attributable to clinical deterioration, treatment discontinuation, or death, with visit-specific counts summarized in **Supplementary Table S4**. The baseline VAS scores were similar between the two groups (LSM, 67.27 in the low-CALLY group vs. 67.09 in the high-CALLY group).

VAS scores decreased from baseline in both groups across all assessed time points, with progressive deterioration over time. A mixed model for repeated measures revealed a significant group-by-time interaction in the LSM change from baseline (P < 0.05). Although no significant between-group differences were observed at earlier assessments (week 6), a marginal, nonsignificant difference favoring the high-CALLY group emerged at week 12 (-1.97 points; 95% CI, -3.97 to 0.04; P = 0.055). This protective effect of a high CALLY index became statistically significant from week 18 onward, as the high-CALLY group showed a significantly less pronounced deterioration in HRQoL compared with the low-CALLY group. Between-group differences in the LSM change from baseline favored the high-CALLY group at week 18 (–3.02 points; 95% CI, –5.28 to –0.76; P = 0.009), week 24 (–4.29 points; 95% CI, –7.32 to –1.25; P = 0.006), and week 30 (–3.75 points; 95% CI, –7.20 to –0.30; P = 0.033). These findings indicated that patients with a high CALLY index experienced a consistently slower decline in HRQoL throughout treatment, with this protective effect reaching borderline significance as early as week 12 and becoming statistically significant from week 18 onward (**Table 2**).

**Table 2:** Longitudinal changes in health-related quality of life assessed by VAS from Baseline.

VAS score	Low-CALLY group		High-CALLY group		Difference in LSM change between groups (95%CI)	P value
	LSM	LSM change from baseline (95% CI)	LSM	LSM change from baseline (95% CI)		
Baseline	67.27	NA	67.09	NA	NA	NA
6 weeks	65.72	-1.55 (-2.40, -0.71)	67.27	-1.82 (-2.66, -0.98)	0.27 (-0.93, 1.46)	0.658
12 weeks	63.83	-3.44 (-4.87, -2.02)	65.61	-1.48 (-2.89, -0.06)	-1.97 (-3.97, 0.04)	0.055
18 weeks	61.05	-6.23 (-7.82, -4.63)	63.88	-3.21 (-4.81, -1.60)	-3.02 (-5.28, -0.76)	0.009
24 weeks	55.26	-12.02 (-14.17, -9.87)	59.36	-7.73 (-9.87, -5.59)	-4.29 (-7.32, -1.25)	0.006
30 weeks	53.15	-14.13 (-16.59, -11.66)	56.72	-10.38 (-12.79, -7.96)	-3.75 (-7.20, -0.30)	0.033

VAS, visual analogue scale; CALLY, CRP-albumin-lymphocyte index; LSM, least squares mean; CI, confidence interval; NA, not applicable.

## 4 Discussion

The management of advanced CCA remains a clinical challenge. The prognosis of advanced CCA is influenced by a complex interplay of factors, including demographic characteristics, diverse treatment backgrounds, tumor biology, patients’ intrinsic factors, and other unknown variables (22–24). The combination of chemotherapy with immune checkpoint inhibitors—chemoimmunotherapy—has become a standard-of-care option for advanced CCA, and recent studies have begun to explore its utility in adjuvant and neoadjuvant settings (25, 26). Although recent advances in chemoimmunotherapy have shown promise for advanced CCA (27), a pressing unmet need persists not only for more effective treatments but also for reliable prognostic biomarkers to guide therapeutic decision-making. In this retrospective PSM cohort study, we demonstrated that the CALLY index—a composite biomarker integrating nutritional status, immune competence, and systemic inflammation—was a significant and independent prognostic factor in patients with advanced CCA receiving chemoimmunotherapy. Specifically, patients with a high CALLY index (>1.42) had significantly longer OS and PFS, along with better preservation of HRQoL.

The prognostic value of the CALLY index aligns with and extends previous findings by Iida et al. (20), who first proposed this composite biomarker in hepatocellular carcinoma. Since its introduction, the CALLY index has been applied across multiple malignancies, including digestive, respiratory, and urinary system cancers (28–30), as well as in nononcologic conditions such as cardiovascular and infectious diseases (31–33). The biological plausibility of the index derives from the established roles of its constituent components in cancer pathophysiology. Serum albumin reflects nutritional status and synthetic function, with low levels suggesting cachexia or inflammation-mediated dysfunction (34). Lymphocytes are central to adaptive immunity and critically contribute to antitumor surveillance (35). In the context of immunotherapy, a high peripheral lymphocyte count may reflect not only robust systemic immune competence but also a less immunosuppressive tumor microenvironment. Recent evidence further supports this dynamic host-tumor interplay. A study has demonstrated that clonally expanded T cells within tumors can be detected in peripheral blood, with their abundance strongly correlated with intratumoral levels (36). Moreover, another report showed that tumor-specific T-cell clones mediating pathological response after PD-1 blockade tend to undergo expansion in peripheral blood, and this peripheral expansion is associated with improved response to immune checkpoint inhibition (37). Collectively, these findings suggest that peripheral lymphocyte abundance may reflect not only overall immune competence but also a tumor microenvironment more permissive to immune infiltration and cytotoxic engagement. However, it should be noted that the absolute lymphocyte count alone may not fully capture the functional state or tissue distribution of these immune cells. Therefore, while the absolute lymphocyte count, as used in the CALLY index, may serve as an accessible indicator that offers insights into both systemic immune competence and potential tumor–immune engagement, it should be interpreted as one component of a more complex immunological picture. Meanwhile, CRP, a marker of systemic inflammation, is elevated in pro-tumor inflammatory states associated with disease progression and poorer differentiation (38). By integrating these three interrelated biological domains, the CALLY index provides a composite measure of host status that is especially pertinent to outcomes in patients undergoing chemoimmunotherapy. We note that although the absolute lymphocyte count was used in accordance with the original CALLY index formulation and common immuno-oncology practice, it can be influenced by factors such as hydration status and inter-laboratory variability. Therefore, future studies exploring normalized ratios may offer additional refinement.

Our analysis revealed a clinically meaningful survival benefit among patients with a high CALLY index, with median OS of 13.00 months versus 11.50 months in the low-CALLY group and median PFS of 7.50 months versus 6.00 months. Following multivariate adjustment, these differences corresponded to a 32.4% reduction in the risk of death and a 27.8% reduction in the risk of disease progression. The CALLY index remained an independent prognostic factor after adjustment for age, treatment line, and ECOG performance status. The robustness of these findings was further supported by comprehensive internal validation, which demonstrated good discriminative performance with optimism-corrected C-indices of 0.704 for OS and 0.716 for PFS. Although these values did not represent exceptional predictive accuracy, they indicated clinically meaningful and acceptable performance within the context of this retrospective study and the complex treatment background of the cohort. The validation also confirmed adequate calibration and fulfillment of the proportional hazards assumption. Collectively, these findings underscore the potential of the CALLY index as a practical tool to enhance risk stratification and guide personalized treatment strategies. Its consistent performance across univariate, multivariate, and internal validation analyses reinforces the robustness of our results and suggests that the biological domains captured by this index represent key determinants of clinical outcome in this patient population.

HRQoL outcomes provide supportive evidence of the clinical relevance of the CALLY index. A significantly slower deterioration in the VAS was observed in the high-CALLY group, becoming statistically significant from week 18 onward. Notably, this VAS was collected as a brief, single-item overall QoL indicator to enhance longitudinal compliance in this real-world cohort, rather than as a pain-specific instrument; therefore, it captures limited domain-level HRQoL information and should be interpreted cautiously. This suggests that the biologically favorable status reflected by this biomarker translates into a modest difference in patients’ global subjective health status, although the observed ~3–4-point separation may not necessarily meet commonly cited minimal important difference thresholds and thus should not be over-interpreted as definitively clinically meaningful. These effects may arise from improved treatment tolerability and more sustained disease control—both of which are plausible and directionally consistent with the immunonutritional advantages captured by the index. Patients with a robust immunonutritional profile may be better equipped to tolerate treatment-related toxicities, thereby helping preserve physical function and reducing symptom burden, while prolonged disease control helps minimize cancer-related symptoms. This time course is consistent with (but does not prove) the typical response pattern seen with immuno-oncology agents, in which clinical benefits often accumulate after the initial treatment cycles. The preservation of quality of life is especially critical in advanced cancer care, where maintaining function and symptom control constitute major treatment goals alongside survival. Taken together, we regard the VAS findings as supportive and hypothesis-generating; further confirmation using multidimensional HRQoL instruments is warranted.

Clinically, our findings suggest several potential applications for the CALLY index, primarily in the realms of prognostication and patient stratification. First, it may help identify patients who are most likely to benefit from chemoimmunotherapy, thereby informing treatment selection. Second, the index may serve as a dynamic monitoring tool during treatment; serial measurements could provide early indications of response or toxicity before radiological or clinical changes become evident. Third, the CALLY index could help stratify patients in clinical trials, ensuring a more balanced distribution of prognostic factors across treatment groups. Furthermore, the findings from this observational study may offer preliminary insights for future investigations into supportive care for patients with a low CALLY index. Potential exploratory directions could tentatively include nutritional optimization, management of inflammation, or strategies aimed at supporting lymphocyte homeostasis. It must be clearly stated that the CALLY index and its constituents should be viewed primarily as an investigational tool at this stage, rather than as established therapeutic targets. Our findings suggest that the host factors captured by this index could inform risk stratification models. Whether modification of these underlying conditions can meaningfully influence clinical outcomes remains to be validated in future, well-designed interventional trials.

Several limitations of our study should be acknowledged. First, its retrospective, single-center design may introduce selection bias, despite the application of PSM to minimize known confounders. Second, to mitigate potential confounding related to the CALLY index components, we excluded patients with active or chronic viral hepatitis, which may limit the generalizability of our findings in high-prevalence regions. Third, residual confounding may persist because treatment-time biliary events and baseline metastatic burden were not consistently available in a standardized format for matching. Fourth, detailed tumor staging (T and N stage) and histopathological factors such as vascular and perineural invasion could not be reliably incorporated into our model. This limitation stems from the inherent imprecision of radiological staging and biopsy-based diagnosis in our non-surgical cohort, and variations in AJCC staging systems across CCA subtypes. Fifth, molecular data (e.g., IDH1, FGFR2) were incomplete due to restricted insurance coverage for genomic profiling in China during the study period. Finally, although all patients received chemoimmunotherapy, we were unable to fully adjust for variations in later-line treatment regimens. Consequently, residual confounding remains possible, and external validation in independent, multicenter cohorts is warranted to confirm the prognostic utility of the CALLY index.

Future research should address several important questions. First, prospective, multicenter studies are needed to validate these findings and confirm generalizability. Second, assessing dynamic changes in the CALLY index during treatment may help clarify its utility as a monitoring tool for disease course. Third, investigating the biological basis underlying the prognostic performance of the index could improve our understanding of the tumor-host interaction. Finally, the CALLY index could serve as a foundational element for risk stratification in future interventional studies, which may explore whether supportive strategies designed to improve a patient’s underlying immunonutritional status translate into more favorable outcomes.

In conclusion, our study identifies the CALLY index as a robust and independent prognostic biomarker for patients with advanced CCA undergoing chemoimmunotherapy. By integrating nutritional, inflammatory, and immune dimensions into a single easily calculable score, the CALLY index offers a holistic assessment of host status that correlates with both survival and quality of life. Incorporation of this index into existing prognostic models may improve risk stratification and help identify patients most likely to benefit from chemoimmunotherapy, thereby supporting more individualized treatment approaches for this challenging malignancy. As oncology continues to advance toward personalized care, multidimensional biomarkers such as the CALLY index—reflecting the complex interplay between host and tumor—are likely to play an increasingly important role in clinical decision-making.

## Data Availability

The datasets presented in this article are not readily available because of restrictions imposed by the institutional review board, but can be made available by the corresponding author upon reasonable request and with approval from the relevant ethics committee. Requests to access the datasets should be directed to Qian Mei, E-mail: meiqnn@hotmail.com.
