# Supplementary material for: A compass in the challenge: the CALLY index as a prognostic biomarker in advanced cholangiocarcinoma treated with chemoimmunotherapy: a retrospective propensity score-matched cohort study
Source: Front Immunol. 2026 Feb 24;17:1713495. doi: 10.3389/fimmu.2026.1713495 (PMC12971696; doi:10.3389/fimmu.2026.1713495)
Supplement: Supplementary file 1 [file DataSheet1.docx]

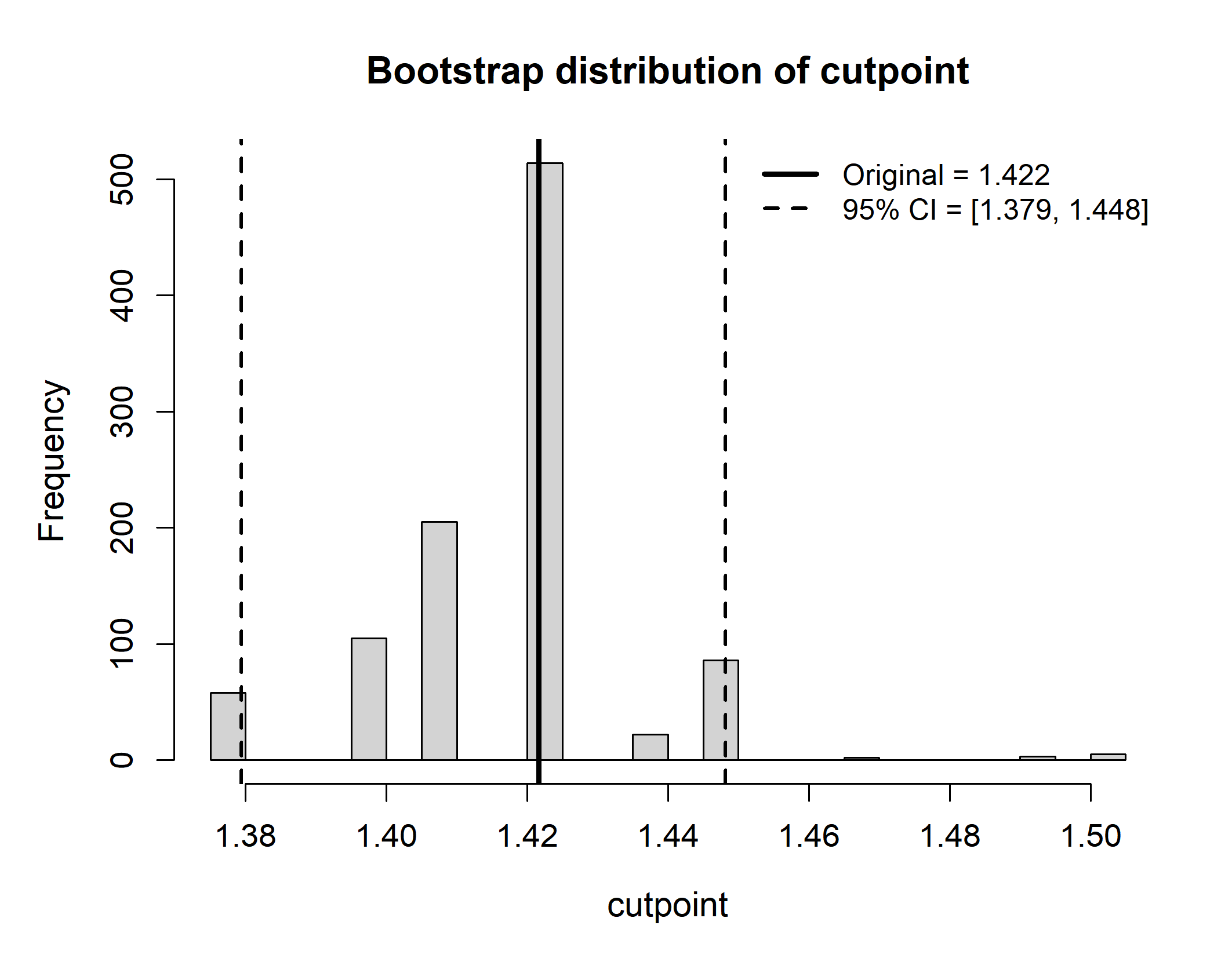


Figure S1. Bootstrap distribution of the optimal CALLY cut-point for overall survival.

The optimal CALLY cut-off point was re-estimated in 1,000 bootstrap resamples (with replacement) from the pre-matched cohort using maximally selected rank statistics (surv_cutpoint). The solid vertical line indicates the cut-point from the original cohort (1.42). Dashed vertical lines indicate the 2.5th and 97.5th percentiles (1.38–1.45), reflecting cut-point stability.


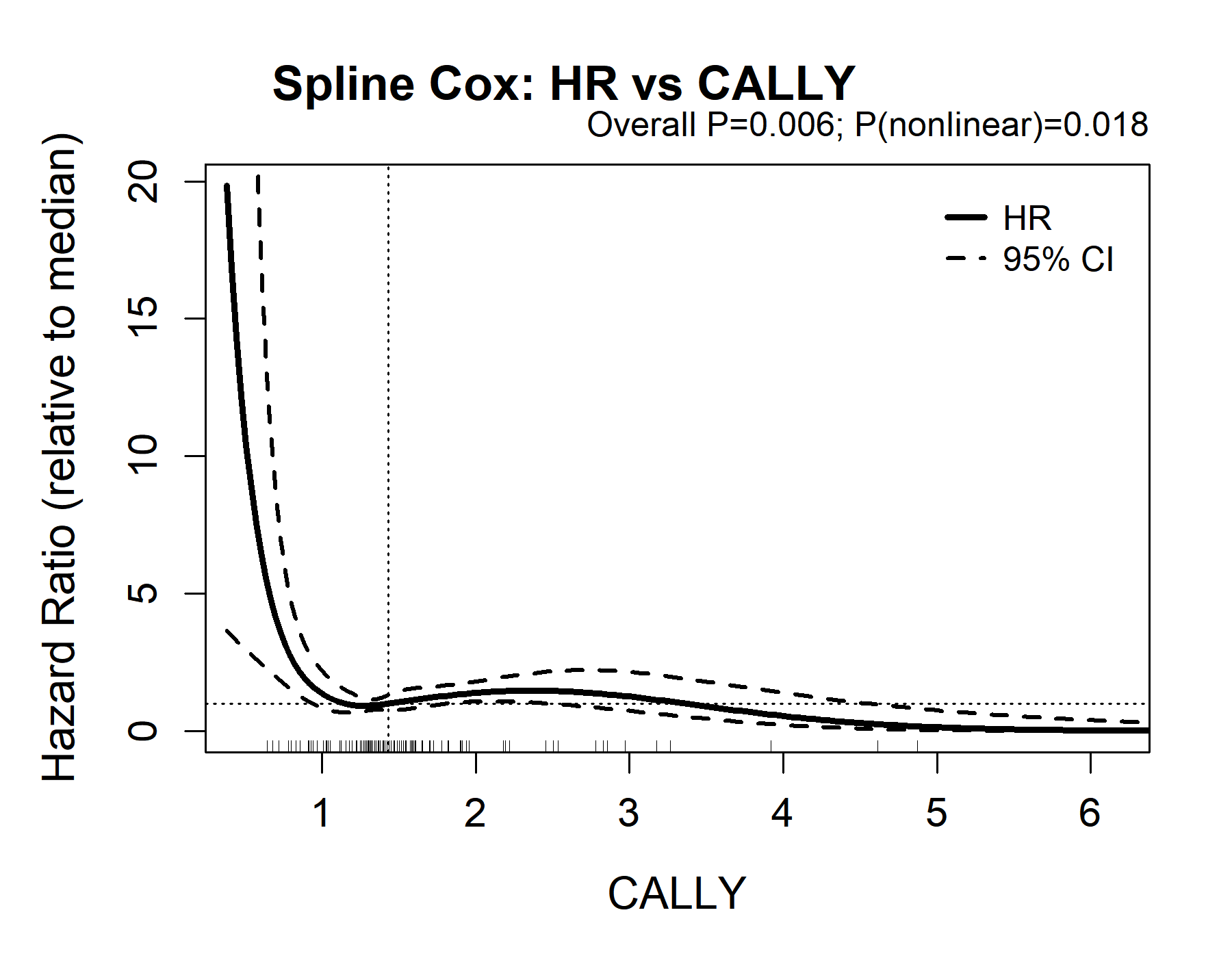


Figure S2. Restricted cubic spline Cox model for the association between continuous CALLY and overall survival in the matched cohort.

Hazard ratios (solid line) and 95% confidence intervals (dashed lines) are shown relative to the median CALLY value (vertical dotted line; HR=1). Rug marks represent the distribution of CALLY. Overall and non-linear P values were obtained from Wald tests of spline terms using matched-pair cluster-robust standard errors (overall P = 0.006; P for nonlinearity = 0.018). CALLY, CRP-albumin-lymphocyte index.


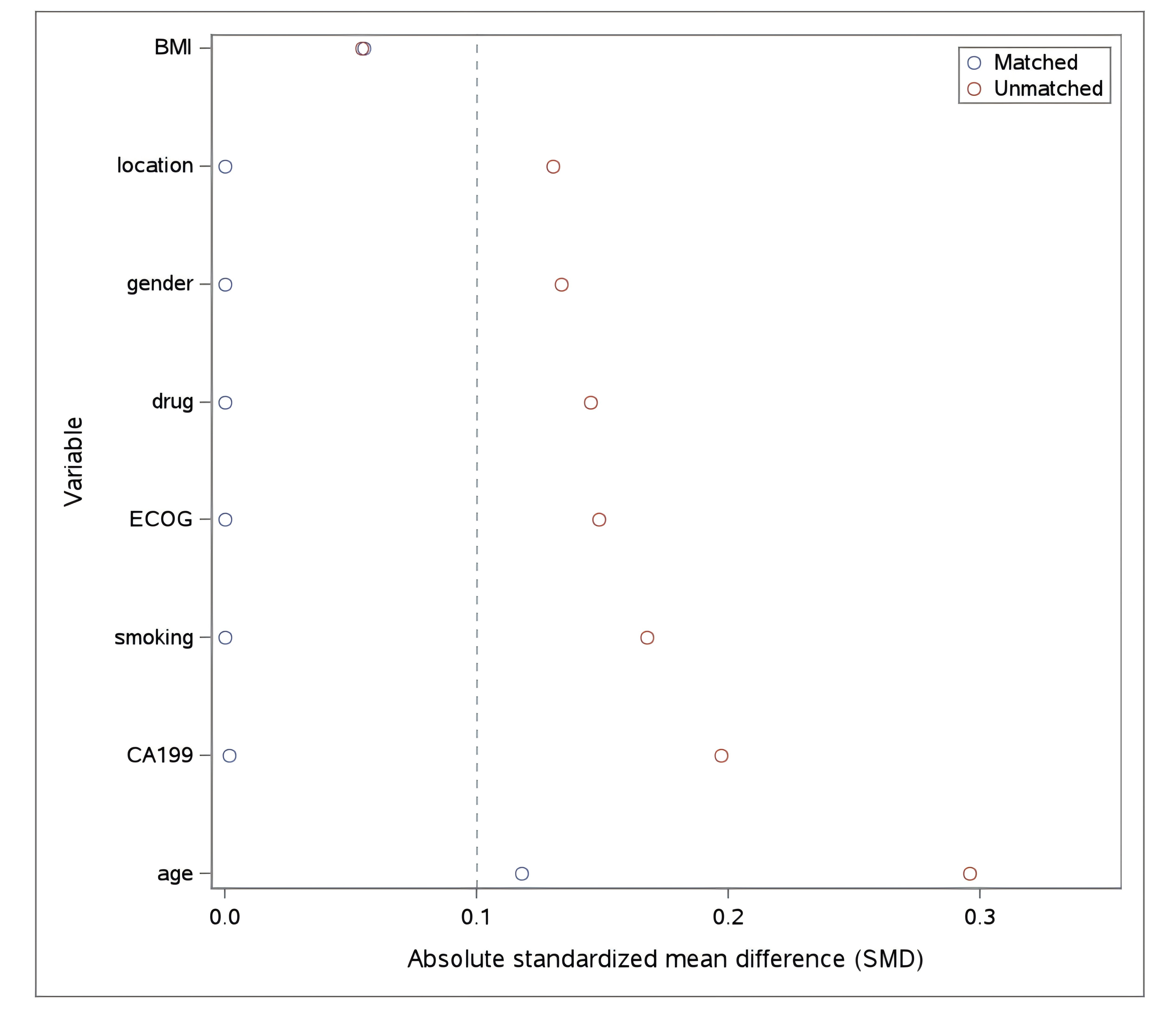


Figure S3. Covariate balance before and after propensity score matching (Love plot).

Absolute standardized mean differences (SMDs) for baseline covariates are shown before matching (Unmatched) and after 1:1 propensity score matching (Matched) between the Low-CALLY and High-CALLY groups. Covariates displayed include age, CA19-9, smoking status, ECOG performance status, treatment line, sex, tumor location, and BMI. The vertical reference line indicates an absolute SMD of 0.10, a commonly used threshold for acceptable covariate balance. After matching, covariate imbalance was substantially reduced, indicating improved comparability between groups.


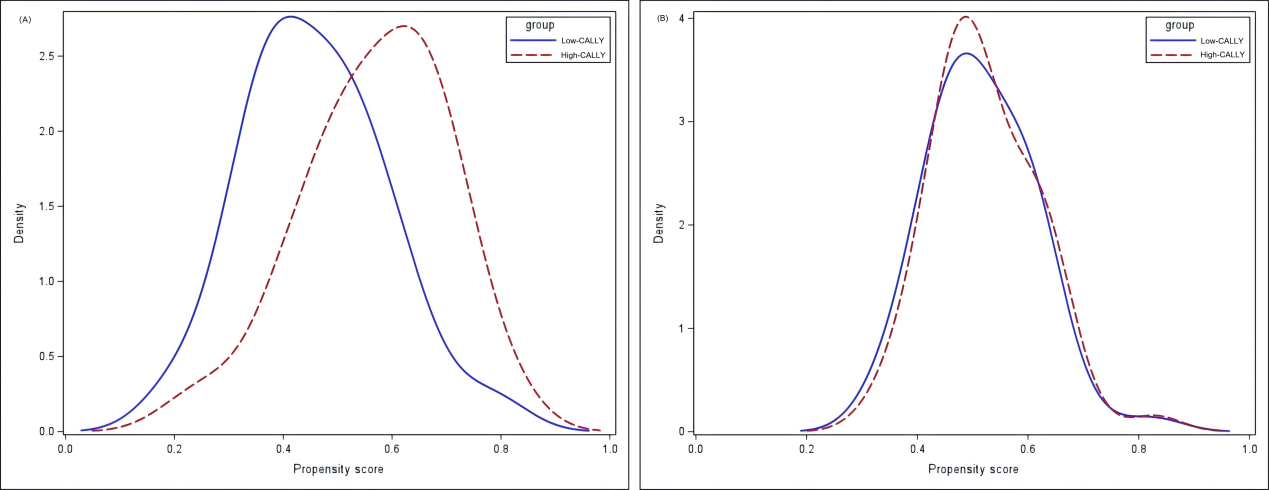


Figure S4. Propensity score overlap (common support) before and after matching.

1. Kernel density distributions of the estimated propensity scores are shown for the Low-CALLY and High-CALLY groups in the unmatched cohort, illustrating the degree of overlap (common support) prior to matching. (B) After 1:1 matching, kernel density distributions of the estimated propensity scores are shown for the Low-CALLY and High-CALLY groups in the matched cohort, demonstrating improved overlap. CALLY, CRP-albumin-lymphocyte index.

| **Quartile** | **term** | **HR** | **LCL** | **UCL** | **P** |
| --- | --- | --- | --- | --- | --- |
| Q1 | Q1 (ref) | NA | NA | NA | NA |
| Q2 | Q2 vs Q1 | 1.18 | 0.55 | 1.95 | 0.275 |
| Q3 | Q3 vs Q1 | 0.71 | 0.39 | 1.30 | 0.229 |
| Q4 | Q4 vs Q1 | 0.62 | 0.32 | 1.24 | 0.178 |
| Trend | per 1 quartile increase | 0.82 | 0.67 | 1.01 | 0.065 |

Table S1. Sensitivity analysis using CALLY quartiles and ordinal trend test in the matched cohort.

CALLY index was categorized into quartiles (Q1–Q4) with Q1 as the reference. Cox models used matched-pair cluster-robust standard errors (clustered by matched pair ID). Trend was tested by modeling quartile rank as an ordinal variable.

| **Variable** | **Total (n=110)** | **Low-CALLY group (n=55)** | **High-CALLY group (n=55)** |
| --- | --- | --- | --- |
| Index regimen start year, median (IQR) | 2022 (2020–2023) | 2021 (2020–2023) | 2022 (2020–2023) |
| Line of therapy at index, n (%) |  |  |  |
| First-line | 52 (47.3) | 26 (47.3) | 26 (47.3) |
| Later-line (≥2nd line) | 58 (52.7) | 29 (52.7) | 29 (52.7) |
| ICI class, n (%) |  |  |  |
| PD-1 inhibitor | 89 (80.9) | 45 (81.8) | 44 (80.0) |
| PD-L1 inhibitor | 21 (19.1) | 10 (18.2) | 11 (20.0) |
| Specific ICI agent, n (%) |  |  |  |
| Pembrolizumab | 82 (74.5) | 42 (76.4) | 40 (72.7) |
| Camrelizumab | 7 (6.4) | 3 (5.5) | 4 (7.3) |
| Durvalumab | 21 (19.1) | 10 (18.2) | 11 (20.0) |
| ICI dosing interval during induction, n (%) |  |  |  |
| Q3W | 110 (100.0) | 55 (100.0) | 55 (100.0) |
| Chemo backbone concurrent with ICI (delivered induction cycles), n (%) [median (IQR) or (range)] **^§^** |  |  |  |
| First-line |  |  |  |
| GP (Q3W) | 50 (45.5) [7 (6–8)] | 25 (45.5) [7 (6–8)] | 25 (45.5) [7 (6–8)] |
| GEMOX (Q3W) | 2 (1.8) [6 (6–8)] | 1 (1.8) [6 (6–6)] | 1 (1.8) [8 (8–8)] |
| Later-line |  |  |  |
| FOLFOX or mFOLFOX (Q2W) | 31 (28.2) [6 (5–7)] | 15 (27.3) [6 (5–8)] | 16 (29.1) [6 (6–6)] |
| FOLFIRI (Q2W) | 9 (8.2) [4 (4–6)] | 4 (7.3) [4 (4–4)] | 5 (9.1) [4 (4–6)] |
| nal-IRI + 5-FU/LV (Q2W) | 7 (6.4) [6 (6–7)] | 4 (7.3) [6 (4–6)] | 3 (5.5) [8 (6–8)] |
| Capecitabine (Q3W) | 6 (5.5) [4 (4–6)] | 4 (7.3) [4 (4–4)] | 2 (3.6) [6 (6–6)] |
| S-1 (Q3W) | 3 (2.7) [4 (4–6)] | 1 (1.8) [6 (6–6)] | 2 (3.6) [4 (4–4)] |
| CAPOX (Q3W) | 2 (1.8) [4 (4–6)] | 1 (1.8) [6 (6–6)] | 1 (1.8) [4 (4–4)] |
| Total ICI cycles, median (IQR) | 14 (10–17) | 12 (9–15) | 15 (13–18) |
| Maintenance strategy after induction, n (%) |  |  |  |
| Any maintenance after induction | 47 (42.7) | 21 (38.2) | 26 (47.3) |
| ICI only | 29 (26.4) | 13 (23.6) | 16 (29.1) |
| ICI + single-agent chemotherapy | 18 (16.4) | 8 (14.5) | 10 (18.2) |
| Subsequent therapies |  |  |  |
| Any subsequent chemotherapy | 51 (46.4) | 23 (41.8) | 28 (50.9) |
| Subsequent chemotherapy (highest line received), n (%) **^†^** |  |  |  |
| One subsequent chemotherapy line (2L only) | 19 (17.3) | 10 (18.2) | 9 (16.4) |
| ≥Two subsequent chemotherapy lines (≥3L) | 32 (29.1) | 13 (23.6) | 19 (34.5) |
| Treatment era **^‡^** |  |  |  |
| Index start year <2021 | 32 (29.1) | 15 (27.3) | 17 (30.9) |
| Index start year ≥2021 | 78 (70.9) | 40 (72.7) | 38 (69.1) |

Table S2. Treatment exposure summary of the index ICI-based therapies in the propensity score-matched cohort.

§ Induction cycles are summarized as median (IQR) when the regimen-specific sample size is ≥10; when sample size is <10, cycles are summarized as median (range) to avoid unstable IQR estimates in small subgroups.

† Subsequent chemotherapy lines were defined relative to the index regimen. Patients were categorized by the highest number of additional chemotherapy lines received after index (one line vs. ≥two lines).

‡ Following updates to international guidelines and local reimbursement, the use of ICI plus chemotherapy increased substantially at our center after 2021.

ICI, immune checkpoint inhibitor; PD-1, programmed cell death 1; PD-L1, programmed death-ligand 1; Q2W, every 2 weeks; Q3W, every 3 weeks; GP, gemcitabine plus cisplatin; GEMOX, gemcitabine plus oxaliplatin; FOLFOX/mFOLFOX, (modified) folinic acid/5-fluorouracil plus oxaliplatin; FOLFIRI, folinic acid/5-fluorouracil plus irinotecan; nal-IRI, nanoliposomal irinotecan; CAPOX, capecitabine plus oxaliplatin; CALLY, CRP-albumin-lymphocyte index.

| **Variable** | **Mean (SD) or n (%)** | **COX for OS** | | | | **COX for PFS** | | | |
| --- | --- | --- | --- | --- | --- | --- | --- | --- | --- |
|  |  | **Univariate analysis** | | **Multivariate analysis** | | **Univariate analysis** | | **Multivariate analysis** | |
|  |  | **HR (95% CI)** | **P value** | **HR (95% CI)** | **P value** | **HR (95% CI)** | **P value** | **HR (95% CI)** | **P value** |
| Age | 62.61 (9.17) | 1.03 (1.01-1.06) | 0.008 | 1.04 (1.02-1.06) | 0.007 | 1.05 (1.03-1.07) | <0.001 | 1.06 (1.04-1.08) | <0.001 |
| Sex |  |  |  |  |  |  |  |  |  |
| Female | 60 (54.55) | 1 | NA |  |  | 1 | NA | NA | NA |
| Male | 50 (45.45) | 0.97 (0.63-1.50) | 0.898 |  |  | 0.97 (0.66-1.44) | 0.888 | NA | NA |
| Smoking |  |  |  |  |  |  |  |  |  |
| No | 38 (34.55) | 1 | NA |  |  | 1 | NA | NA | NA |
| Yes | 72 (65.45) | 1.34 (0.87-2.09) | 0.189 |  |  | 1.08 (0.73-1.60) | 0.696 | NA | NA |
| BMI | 22.83 (3.11) | 0.93 (0.86-1.01) | 0.095 |  |  | 1.00 (0.94-1.05) | 0.877 | NA | NA |
| CA 19-9 | 221.24 (313.23) | 1.00 (1.00-1.00) | 0.395 |  |  | 1.00 (1.00-1.00) | 0.371 | NA | NA |
| Tumor location |  |  |  |  |  |  |  |  |  |
| iCCA | 34 (30.91) | 1 | NA |  |  | 1 | NA | NA | NA |
| eCCA | 76 (69.09) | 1.35 (0.86-2.12) | 0.196 | NA | NA | 1.12 (0.77-1.63) | 0.566 | NA | NA |
| Treatment line |  |  |  |  |  |  |  |  |  |
| Front-line | 52 (47.27) | 1 | NA | 1 | NA | 1 | NA | 1 | NA |
| Later-line | 58 (52.73) | 2.00 (1.24-3.22) | 0.005 | 2.38 (1.36-4.16) | 0.002 | 2.14 (1.42-3.23) | <0.001 | 2.18 (1.50-3.18) | <0.001 |
| ECOG |  |  |  |  |  |  |  |  |  |
| <=1 | 40 (36.36) | 1 | NA | 1 | NA | 1 | NA | 1 | NA |
| >1 | 70 (63.64) | 1.80 (1.14-2.86) | 0.012 | 2.37 (1.36-4.07) | 0.001 | 1.41 (0.94-2.13) | 0.099 | 1.49 (1.02-2.17) | 0.040 |
| CALLY | 1.66 (0.97) | 0.64 (0.48-0.85) | 0.002 | 0.66 (0.48-0.89) | 0.018 | 0.72 (0.61-0.85) | <0.001 | 0.69 (0.57-0.85) | <0.001 |

Table S3. Univariate and multivariate Cox proportional hazards models for overall survival (OS) and progression-free survival (PFS) in the propensity score-matched cohort, stratified by treatment era.

For multivariable models, only variables retained in the final model are reported; cells are left blank where not applicable. OS, overall survival; PFS, progression-free survival; HR, hazard ratio; CI, confidence interval; ECOG, Eastern Cooperative Oncology Group performance status; iCCA, intrahepatic cholangiocarcinoma; eCCA, extrahepatic cholangiocarcinoma; CALLY, CRP-albumin-lymphocyte index; NA, not applicable.

| **Timepoint**  **(Week)** | **Group** | **Expected**  **(n)** | **Completed VAS**  **(n)** | **Completion Rate** | **Compliance Rate** | **Reasons for Missing Data (n)** | | | |
| --- | --- | --- | --- | --- | --- | --- | --- | --- | --- |
|  |  |  |  |  |  | **Clinical Deterioration** | **Refusal** | **Treatment Discontinuation** | **Death** |
| Baseline | Low-CALLY | 55 | 55 | 100.00% | 100.00% | 0 | 0 | 0 | 0 |
|  | High-CALLY | 55 | 55 | 100.00% | 100.00% | 0 | 0 | 0 | 0 |
| 6 | Low-CALLY | 55 | 55 | 100.00% | 100.00% | 0 | 0 | 0 | 0 |
|  | High-CALLY | 55 | 55 | 100.00% | 100.00% | 0 | 0 | 0 | 0 |
| 12 | Low-CALLY | 55 | 53 | 96.36% | 96.36% | 2 | 0 | 0 | 0 |
|  | High-CALLY | 55 | 54 | 98.18% | 98.18% | 1 | 0 | 0 | 0 |
| 18 | Low-CALLY | 55 | 52 | 94.55% | 94.55% | 2 | 1 | 0 | 0 |
|  | High-CALLY | 55 | 51 | 92.73% | 92.73% | 3 | 1 | 0 | 0 |
| 24 | Low-CALLY | 55 | 48 | 90.57% | 87.27% | 3 | 1 | 1 | 2 |
|  | High-CALLY | 55 | 50 | 90.90% | 90.90% | 3 | 1 | 1 | 0 |
| 30 | Low-CALLY | 55 | 44 | 91.67% | 80.00% | 2 | 1 | 1 | 7 |
|  | High-CALLY | 55 | 48 | 90.57% | 87.27% | 2 | 2 | 1 | 2 |

Table S4. Visit-specific evaluable sample size and reasons for missing VAS assessments in the propensity score–matched cohort

Completion rate (%): Completed VAS / (Expected − Death) at that visit, reflecting completion among patients alive at the scheduled assessment (i.e., excluding those who could not be assessed due to death).

Compliance rate (%): Completed VAS / Expected, reflecting overall follow-up compliance including attrition due to death/discontinuation.

Reasons for missing data: Missingness was categorized into clinical deterioration, refusal, treatment discontinuation, and death based on the primary reason documented around the scheduled assessment; categories were treated as mutually exclusive.

CALLY, CRP-albumin-lymphocyte index.
